# Exploring climate change on Twitter using seven aspects: Stance, sentiment, aggressiveness, temperature, gender, topics, and disasters

**DOI:** 10.1371/journal.pone.0274213

**Published:** 2022-09-21

**Authors:** Dimitrios Effrosynidis, Georgios Sylaios, Avi Arampatzis

**Affiliations:** 1 Database & Information Retrieval research unit, Department of Electrical & Computer Engineering, Democritus University of Thrace, Xanthi, Greece; 2 Lab of Ecological Engineering & Technology, Department of Environmental Engineering, Democritus University of Thrace, Xanthi, Greece; Shenzhen University, CHINA

## Abstract

How do climate change deniers differ from believers? Is there any correlation between human sentiment and deviations from historic temperature? We answer nine such questions using 13 years of Twitter data and 15 million tweets. Seven aspects are explored, namely, user gender, climate change stance and sentiment, aggressiveness, deviations from historic temperature, topics discussed, and environmental disaster events. We found that: a) climate change deniers use the term global warming much often than believers and use aggressive language, while believers tweet more about taking actions to fight the phenomenon, b) deniers are more present in the American Region, South Africa, Japan, and Eastern China and less present in Europe, India, and Central Africa, c) people connect much more the warm temperatures with man-made climate change than cold temperatures, d) the same regions that had more climate change deniers also tweet with negative sentiment, e) a positive correlation is observed between human sentiment and deviations from historic temperature; when the deviation is between −1.143°*C* and +2.401°*C*, people tweet the most positive, f) there exist 90% correlation between sentiment and stance, and -94% correlation between sentiment and aggressiveness, g) no clear patterns are observed to correlate sentiment and stance with disaster events based on total deaths, number of affected, and damage costs, h) topics discussed on Twitter indicate that climate change is a politicized issue and people are expressing their concerns especially when witnessing extreme weather; the global stance could be considered optimistic, as there are many discussions that point out the importance of human intervention to fight climate change and actions are being taken through events to raise the awareness of this phenomenon.

## Introduction

Over the latest decades, social media platforms allow users a wide spectrum of digital interaction and information exchange, producing overwhelming sets of user-generated Big Data [1]. Environmental management and sustainability topics have been widely analyzed with Big Data Analytics techniques to reveal hidden trends and patterns in public perceptions, mostly utilizing data from the Twitter microblogging service [2–4]. The impact of climate change becomes more evident to society and the economy at scales ranging from global to local. The general public becomes acquainted with scientific evidence indicating that the continuous rise in the Earth’s mean temperature is related to anthropogenic emissions of greenhouse gases, like carbon dioxide, methane, and nitrous oxides [5].

Several aspects of climate change through human opinions have been studied over the past years by researchers. The pioneering work of [6] initiated a research agenda on analyzing the semantics of conversations in social media regarding energy and climate change. They found that tweets allow us to describe behaviors, latitudes, and even to distinguish opinions of the general public. The first study of climate change on Twitter was by [7], where they considered whether important events affect the discussion, finding large variations across metropolitan areas and by topic. At the same period [8], identified three groups of human stance towards climate change, namely supportive, unsupportive and neutral [9], found that messages between like-minded users typically carry positive sentiment, while skeptics and activists carry negative sentiment, and [10] identified the different levels of positive and negative opinions hidden in society. In addition [11], found that climate change skeptics use sarcasm and incivility in their tweets and [12] that anti-climate change tweets are largely not credible. Furthermore, the psychological impacts of climate change like increased aggressiveness and conflicts were examined by [13].

The first works that used machine learning in the manner of sentiment classification are [14, 15], were it was discovered that major climate events can result in a sudden change in sentiment polarity and natural disasters contribute to a decrease in the level of overall happiness. In more recent years [16], used Twitter to identify whether a tweet is relevant or not to a hurricane [17], classified Twitter users into climate change believers and non-believers [18], analyzed the polarity in the expressed opinions [19], reported correlation between human sentiment and both hot and cold temperatures, and [20] compared the sentiment and emotion of climate change tweets between two countries.

A common topic of interest is the comparison of the keywords ‘climate change’ and ‘global warming’ [21–23], where it was found that ‘global warming’ is particularly associated with hoaxes and correlated with anomalous temperature. Another topic of study is how the two genders express their opinions on climate change [20, 24], where it was found that while both genders use very similar language, female tweeters had a convinced attitude towards the anthropogenic impact on climate change, while male tweeters presented a skeptical stance. Finally, another aspect of concern for many researchers is the topics discussed in climate change related tweets [16, 25].

Extreme weather and disaster events are often studied in regards to climate change and result in increased Twitter activity [16, 26–30]. It has been showed that: the spatial distribution of the extreme event tweets is significantly different from the off-topic tweets [31]; flooding was by far the most tweeted topic in connection to climate change, illustrating a strong spiking activity triggered by extreme events [29]; the public recognizes extreme temperature anomalies and attributes them to climate change [32]; society rejects the existence of climate change even when extreme events happen [33]; Twitter activity is highly correlated with the economic damage caused by disaster events [34]; weather events can cause immediate attention to climate change [35]; not all events cause the same attention [36, 37].

Our work delivers several contributions to all the research effort that has been made in the field of climate change and Twitter. Past works use few aspects of climate change, which are not generated with state-of-the-art machine learning methods and address the subject from limited perspectives. For example, they only study the sentiment of the tweets for one year at specific locations and with a limited number of tweets to draw concrete conclusions. In contrast, we explore seven dimensions of climate change via Twitter texts namely, stance, sentiment, aggressiveness, temperature, gender, topics and disasters, and their interactions. On top of that we are using the most comprehensive and quality climate change Twitter dataset to date in both temporal coverage (over 13 years) and volume (over 15 million tweets), where its seven dimensions were produced using the most competitive, state-of-the-art machine learning algorithms. Combined, we are able to study climate change and human opinions in a multi-dimensional perspective and get a thorough view of this large-scale geophysical phenomenon that torments society.

More specifically, we will answer the following 9 research questions:

1. How do climate change deniers differ from believers?
2. Where around the globe the climate change denier/believer ratio is high?
3. Is there any correlation between climate change denier/believer ratio and deviations from historic temperature?
4. What is the sentiment of climate change tweets across the world?
5. Is there any correlation between human sentiment and deviations from historic temperature?
6. How are sentiment, stance, and aggressiveness related regarding climate change?
7. Are the sentiment about climate change and the denier/believer ratio correlated with disaster events based on total deaths, number of affected, and damage costs?
8. What topics are discussed in climate change/global warming discourses on Twitter?
9. Is there any correlation between each of the discovered topics and the climate change stance, sentiment, and aggressiveness?

## Materials and methods

This work is based on the Climate Change Twitter dataset of our previous work [38], which is publicly available (https://data.mendeley.com/datasets/mw8yd7z9wc/). It is the result of the merging of three Twitter climate change datasets (Credibility of Climate Change Denial in Social Media [12], Climate Change Tweets IDs [39], and Internet Archive (https://archive.org/details/twitterstream)), which were further pre-processed and enriched. The enrichment included several tools, external datasets, and state-of-the-art machine learning algorithms accompanied by evaluation to confirm their integrity. The dataset associates each tweet with seven dimensions, which are described in detail in the paper that introduces the dataset. Briefly, these seven dimensions or aspects of the climate change are:

1. Geolocation: That is the longitude-latitude pair the tweet was written.
2. Climate change stance: That is if the tweet supports the belief of man-made climate change (believer), if the tweet does not believe in man-made climate change (denier), and if the tweet neither supports nor refuses the belief of man-made climate change (neutral).
3. Climate change sentiment: A score on a continuous scale. This scale ranges from -1 to 1 with values closer to 1 being translated to positive sentiment, values closer to -1 representing a negative sentiment while values close to 0 depicting no sentiment or being neutral.
4. Climate change aggressiveness: That is if the tweet contains aggressive language or not.
5. Gender: Whether the user that made the tweet is male, female, or undefined.
6. Deviations from historic temperature: The temperature deviation in Celsius and relative to the January 1951-December 1980 average at the time and place the tweet was written.
7. Climate change discussed topics: Categorization of the tweet in one of ten topics namely, seriousness of gas emissions, importance of human intervention, global stance, significance of pollution awareness events, weather extremes, impact of resource overconsumption, Donald Trump versus science, ideological positions on global warming, politics, and undefined.

**Fig 1:**
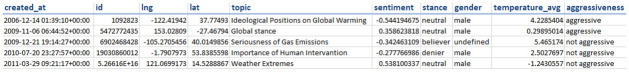
A sample of five rows of the Climate Change Twitter Dataset. The dimensions of geolocation (lng, lat), topic, sentiment, stance, gender, temperature deviation from historic average and aggressiveness are present.

On top of the seven dimensions there exists a dataset of 4,913 natural disasters that occurred during the covered period. A sample of the Climate Change Twitter Dataset is visible in Fig 1.

**Fig 2:**
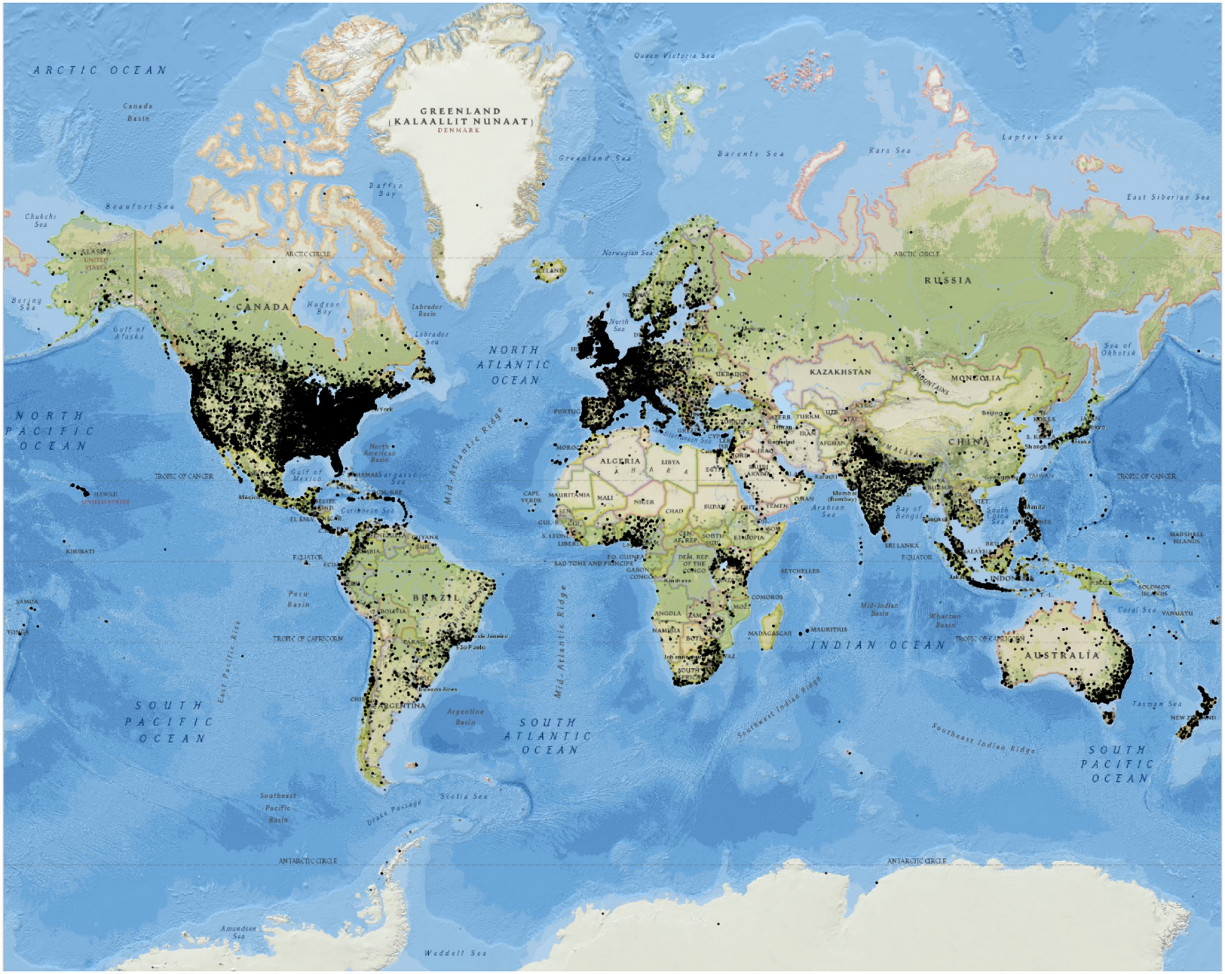
Spatial distribution of the climate-change tweets. Each dark dot is a tweet on the world map.

The data is consisted of 15,789,411 tweets spanning 13 years, from June 6, 2006, to October 1, 2019. One third of them (5,307,538) has geolocation information. The spatial distribution of the tweets can be seen in Fig 2. Since the keywords and hashtags used to create the dataset are in the English language, there is a bias on the origin locations of the tweets. Most of the tweets are located in the United States of America, Canada, United Kingdom, Australia, New Zealand, but also in Europe, where English is very common as a second language. Countries with high populations like India, Japan, the Philippines, Indonesia, and Malaysia have also a high number of English tweets. Tweets in China are almost absent because the country has blocked Twitter. Other locations with tweet activity are the Gulf of Guinea, Uganda, Kenya, South Africa, Central America, and the East coast of Brazil.

There are three potential biases in the data. The queries used to retrieve them are in English, the data is a merge of three datasets, and the volume of tweets is increasing over time as Twitter grows in popularity. In order to deal with the biases, all our analyses will be proportional and not absolute.

## Results and discussion

### Climate change and stance

In this section, we will answer three research questions: a) How do climate change deniers differ from believers? b) Where around the world the climate change denier/believer ratio is high? c) Is there any correlation between climate change denier/believer ratio and deviations from historic temperature?

**Fig 3:**
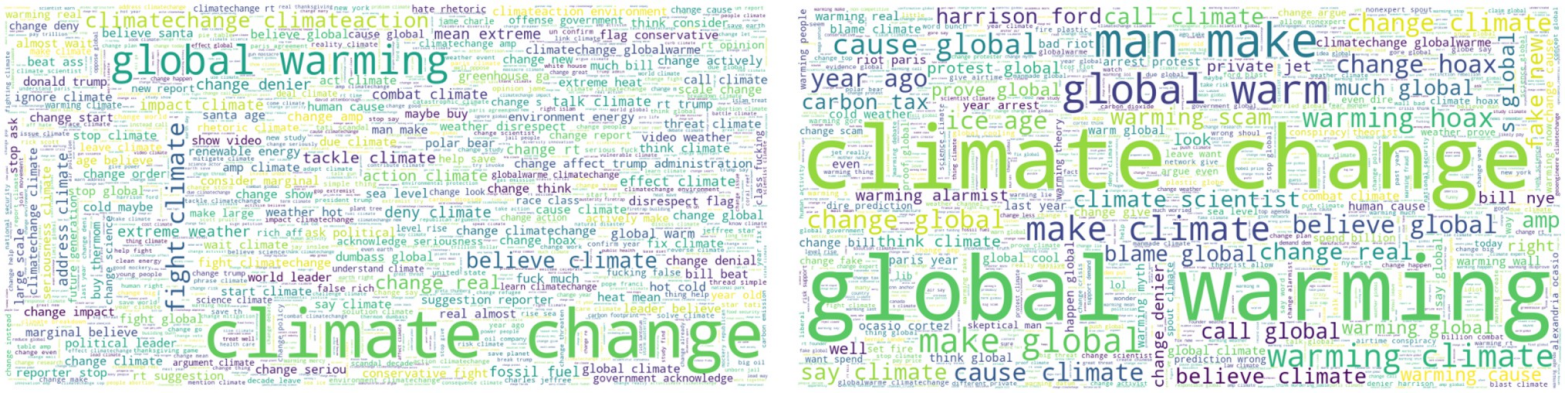
Word clouds of tweets classified as supporting man-made climate change (left) and denying man-made climate change (right). Unigrams/bigrams with a bigger font size have a greater frequency.

To begin with the first question, the 1,000 most common words used by believers and deniers can be seen in Fig 3. Additionally, on the upper part of Table 1, the top-20 unigrams and top-10 bigrams are shown. A major difference between believers and deniers is on the use of ‘global warming’ versus ‘climate change’. Believers use the term ‘global warming’ 5 times less often than ‘climate change’, while deniers favor ‘global warming’. Top words and phrases used by believers that are not used by deniers include the following: ‘believe’, ‘trump’, ‘fight’, ‘stop’, ‘think’, ‘say’, ‘action’, ‘help’, and ‘fight climate’, ‘believe climate’, ‘change real’, ‘do believe’, ‘tackle climate’, ‘action climate’, ‘change denier’, and ‘address climate’. On the other side, deniers use more the following unigrams and bigrams: ‘make’, ‘man’, ‘scientist’, ‘cause’, ‘warm’, ‘snow’, ‘weather’, ‘hoax’, ‘gore’, ‘cold’, and ‘man make’, ‘make climate’, ‘warming climate’, ‘cause global’, ‘warming hoax’, ‘cause climate’, and ‘ice age’. From the above unigrams/bigrams, users classified as believing in man-made climate change, support that using appropriate words and tweet more about taking actions to fight it. Users classified as not believing in man-made climate change tweet more about global warming and extreme weather events.

**Table 1:** Most common words between climate change believers and deniers from all users, females and males.

	Believers (71.51%)		Deniers (7.54%)	
unigrams	climate, change, global, warming, do, believe, people, trump, fight, world, year, new, real, stop, think, say, action, time, science, help		global, climate, warming, change, do, make, year, man, scientist, cause, science, people, warm, snow, weather, hoax, gore, world, real, cold	
bigrams	climate change, global warming, fight climate, believe climate, change real, do believe, tackle climate, action climate, change denier, address climate		global warming, climate change, man make, global warm, make climate, warming climate, cause global, warming hoax, cause climate, ice age	
	Females (73.56%)	Males (70.83%)	Females (5.98%)	Males (8.38%)
unigrams	climate, change, global, warming, do, believe, trump, people, fight, world, year, real, stop, new, think, action, say, time, help, weather	climate, change, global, warming, do, world, fight, believe, people, trump, new, year, real, science, think, action, say, stop, time, environment	global, climate, warming, change, make, do, year, man, scientist, science, cause, say, people, snow, believe, warm, weather, gore, think, hoax	global, climate, warming, change, do, year, make, man, scientist, cause, science, cause, science, people, say, warm, believe, snow, weather, think
bigrams	climate change, global warming, fight climate, believe climate, change real, mean extreme, do believe, extreme weather, tackle climate, action climate	climate change, global warming, fight climate, believe climate, change real, tackle climate, do believe, change denier, action climate, address climate	global warming, climate change, man make, global warm, make global, make climate, warming climate, cause global, warming hoax, believe global	global warming, climate change, man make, global warm, make, climate, make global, warming climate, cause global, warming hoax, year ago

The percentage that each group represents of the total tweets is in parentheses.

Climate change believers are about 10 times more than deniers (71.51% versus 7.54%), while the rest 20.95% are neutral. If we further split the groups into females and males it is evident that the climate change belief percentage of females is higher than males by 3% and the denier percentage of females is by 3% lower than males. Concerning the language used by males and females, our results are in line with the bibliography, as there are no significant differences between the two genders [24]. The only difference that we find, based on the top phrases used, is that female believers are more sensitive to extreme weather events.

Finally, a major difference between deniers and believers is observed in the aggressiveness of their tweets. By classifying each tweet to whether it contains aggressive text (score is 1) or non-aggressive text (score is 0), tweets of believers have an average of 0.27 aggressiveness score, while deniers have 0.42 (neutral users have 0.28).

**Fig 4:**
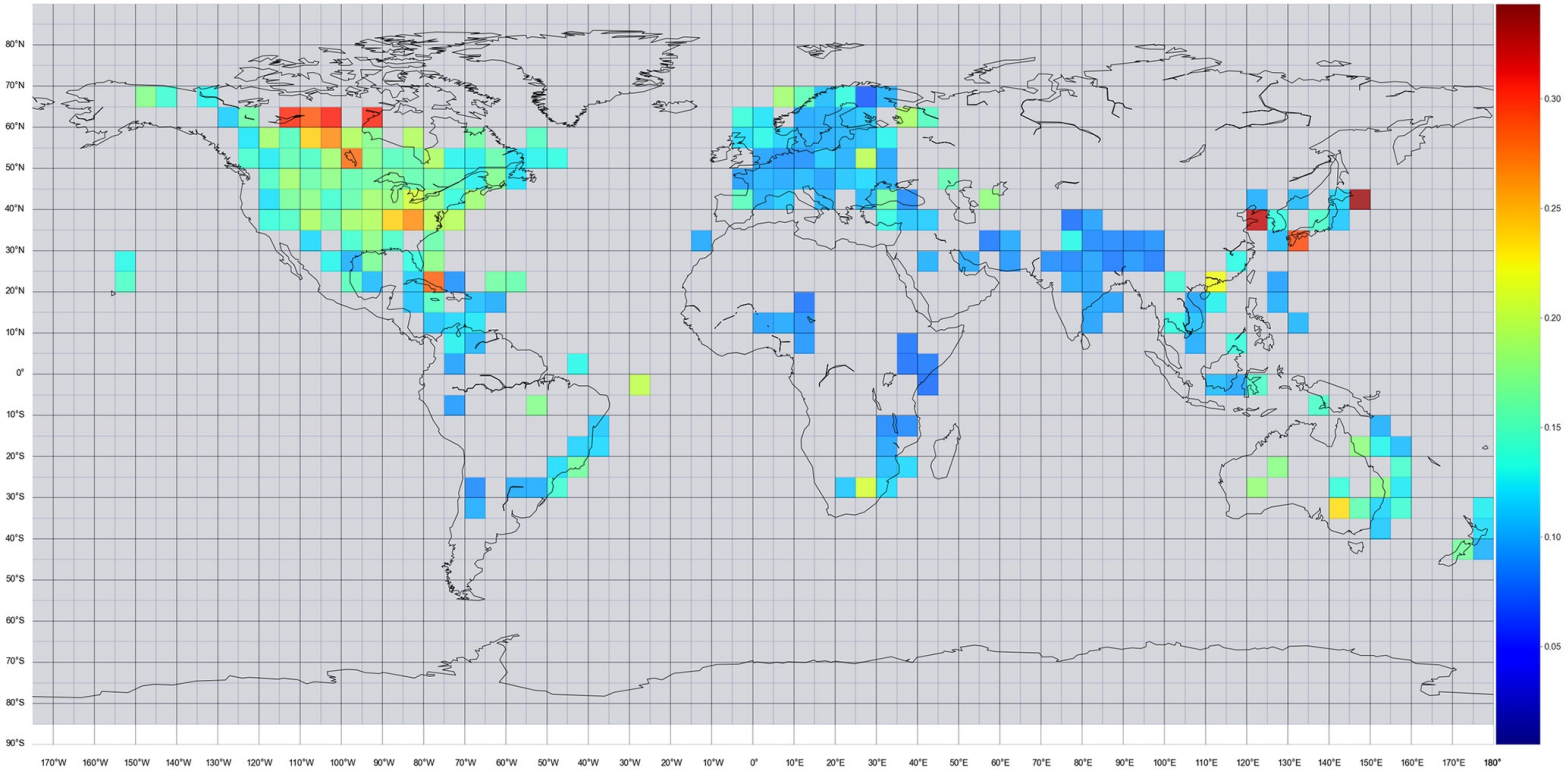
Denier/believer ratio on 5 × 5 arc-minute grid, where there are at least 20 deniers. The ratio ranges between 0 (dark blue) to +0.35 (dark red).

We can split the world map into 5 × 5 arc-minute cells and compute the average denier/believer ratio of all tweets that fall inside each cell. In order to exclude outliers, we will only do this computation if the number of climate change denier tweets is at least 20. The result is visible in Fig 4. Interpreting this map can show where deniers are more present. Canada, the United States of America, Cuba, South Africa, Japan, Eastern China, Australia, and Brazil have much more climate change deniers than the rest of the world. On the contrary, Europe, India, and Central Africa have low denier/believer ratios.

Our results can be explained if we consider the outcomes of the December 2019 UN Climate Change Conference (COP25). In this global conference of high importance, countries such as the United States of America, Brazil, and Australia blocked significant action (see https://www.nytimes.com/2019/12/15/climate/cop25-un-climate-talks-madrid.html). This, combined with the fact that one month before the conference, President Trump formally confirmed the exit of the United States from the Paris agreement, has created a so called ‘Trump effect’ [40]. Since the United States is one of the most powerful countries in the world and its president receives mass media coverage, many people, especially in North America, but also in the rest of the world, are being affected. According to the Spring 2018, Global Attitude Survey [41], people of Canada, Australia, United States of America, and South Africa express low levels of climate change concern, with 66%, 60%, 59%, 59% of them respectively labeling climate change a major threat. As a comparison, other countries on the list like Greece, South Korea, and France are more likely to be concerned about climate change, with 90%, 86%, and 83% respectively seeing it as a serious threat. Diving even further in the same survey, it is interesting to see how 59% of Americans that believe in the threat of climate change are distributed among Republicans and Democrats. Only 27% of Republicans see climate change as a major threat, compared with 83% of Democrats. This polarization and the ‘Trump effect’ might exist and affect other countries sharing the same political opinions.

**Fig 5:**
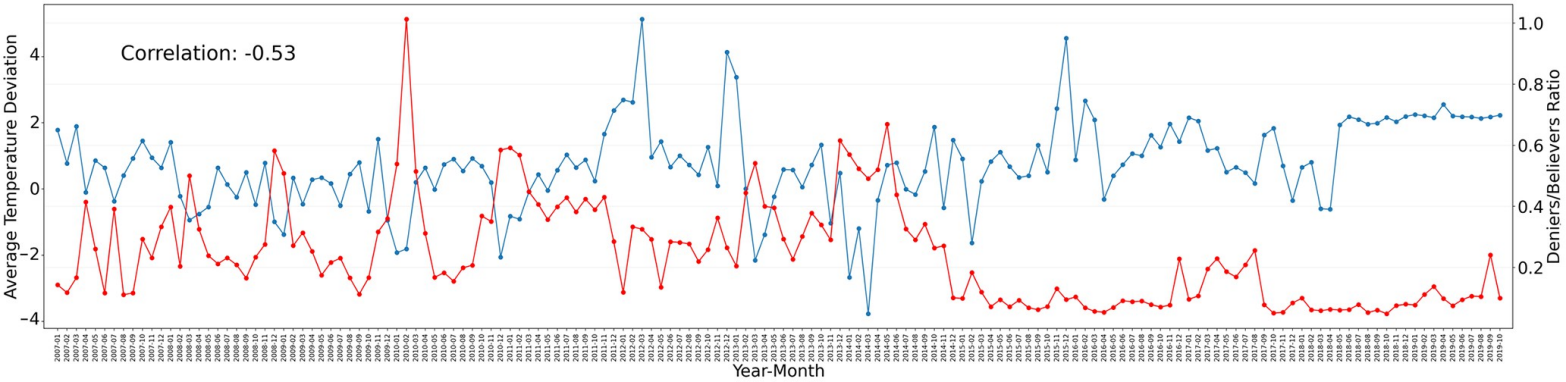
Average historic temperature deviation versus climate change denier/believer ratio over time. On the left y-axis, the average temperature deviation per month is shown (blue). On the right y-axis, the ratio of climate change deniers/believers per month is displayed (red). The correlation between the two lines is also depicted on the top-left.

In order to answer whether there is a correlation between the denier/believer ratio and deviations from historic temperature, we can look at Fig 5. The blue line represents the average temperature deviation at the geolocations where tweets were posted in that month (average of the month), and spans from −4°*C* to + 5°*C*. The red line represents the denier/believer ratio at the geolocations where temperature records are available. The mean of the temperature deviations is +0.73, indicating a temperature rise from the 1950–1980 average. There is volatility in deviations for all months in the studied period, except May 2018 and onwards, where there is a steady +2°*C* temperature deviation from the historic average. The hottest months were March 2012 (+5.1°*C*), December 2012 (+4.1°*C*), and December 2015 (+4.6°*C*), while the coldest month was March 2014 (−3.8°*C*). A negative correlation is evident between deviations from historic temperature and the denier/believer ratio. Using Pearson correlation, the value is -0.53. When the temperature rises compared to the historic average in a particular month, climate change believers usually overtake deniers in Twitter climate change discussions.

**Fig 6:**
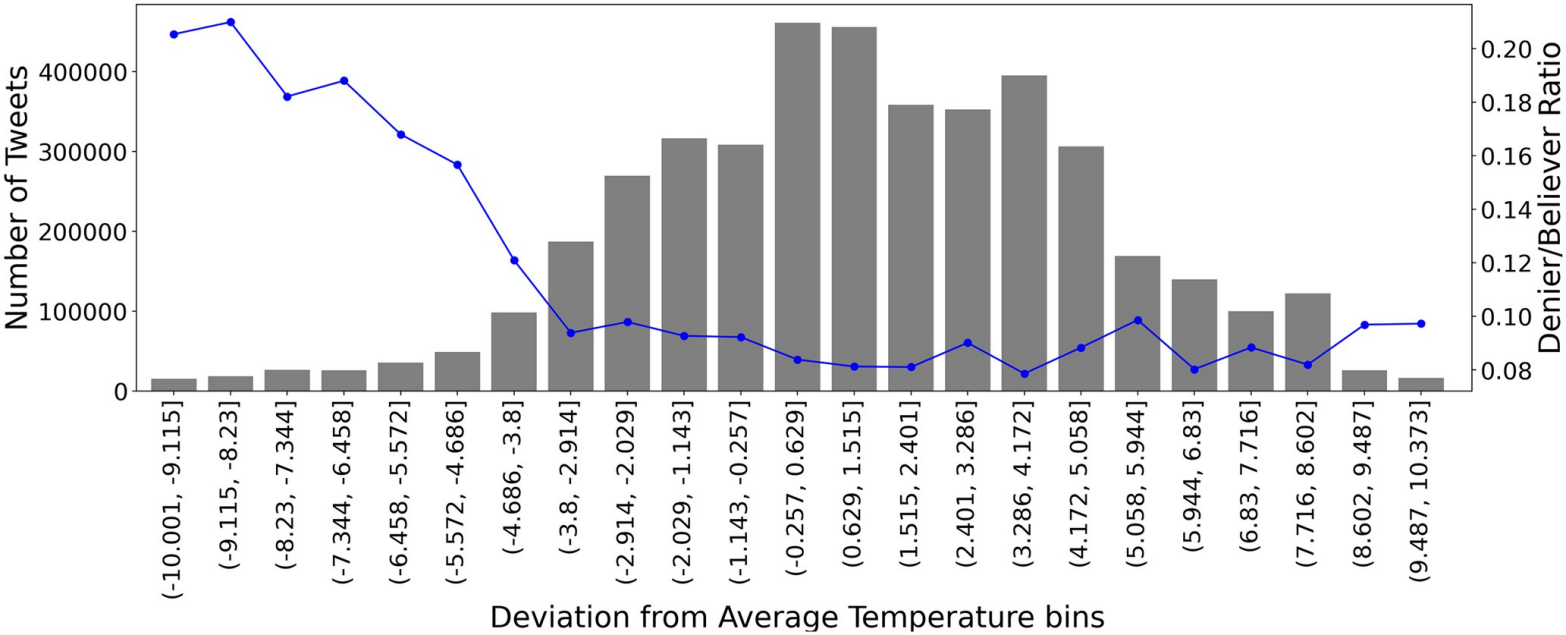
Deviation from average temperature after creating 50 equal bins versus the number of tweets (grey, left y-axis) and denier/believer ratio (blue, right y-axis). Only bins between -10 and +10 temperature deviation are shown as the others contain significant few numbers of tweets, abnormal temperatures, and can be considered outliers.

We can take the temporal variable out of the equation and examine further into the pure correlation between temperature and the denier/believer ratio. For that purpose, we formed 50 equal-sized bins from temperature deviations. Values lower than −10°*C* and higher than +10°*C* can be considered outliers since these are extreme abnormalities in temperature and the volume of tweets in these bins is very low. Excluding these bins, we get the Fig 6. The starting and ending point of the bins are visible on the x-axis. The amplitude of the bars signifies the total number of tweets posted in temperature deviations within the bin. The blue line with points represents the denier/believer ratio in that bin. Several conclusions can be drawn. When extreme lower temperatures are experienced (below −3.8°*C*), climate change deniers are much more present than they usually are. They fade as the temperature falls between normal levels (between −3.8°*C* and −0.257°*C*), reaching their lower at temperatures between −0.257°*C* and +4.172°*C*. When extreme higher temperatures are experienced, deniers rise, but much less. This indicates that people connect much more warm temperatures with man-made climate change than cold temperatures.

### Climate change and sentiment

In this section, we will answer four research questions: a) What is the sentiment of climate change tweets across the world? b) Is there any correlation between human sentiment and deviations from historic temperature? c) How are sentiment, stance, and aggressiveness related regarding climate change? d) Are the sentiment about climate change and the denier/believer ratio correlated with disaster events based on total deaths, number of affected and damage costs?

**Fig 7:**
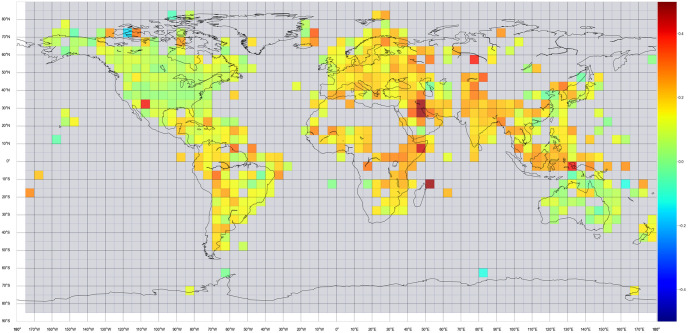
Average sentiment on 5 × 5 arc-minute grid, where there are at least 20 tweets. Sentiment ranges from -0.5 (dark blue), to +0.5 (dark red).

To answer the research questions, we begin with Fig 7, where the world map is split in 5 × 5 arc-minute cells, each of them colored based on its average sentiment. To exclude outliers, only cells containing at least 20 tweets are displayed. Some of the same insights as the ones observed with the denier/believer world map of the previous section also apply here. The most noticeable insight is the case of North America and Oceania. Twitter users of both of these regions are mainly negative about climate change. These same regions presented high levels of climate change deniers as we also saw in our previous analysis. In the same manner, Brazil, South Africa, Japan, Eastern China, and the United Kingdom are characterized by negativity in their climate change tweets. On the other side of the coin, India, Europe, Middle East, and southeast Asia are tweeting positively about climate change. So we can see a positive correlation between denying that the source of climate change is humans and tweeting with negative sentiment.

**Fig 8:**
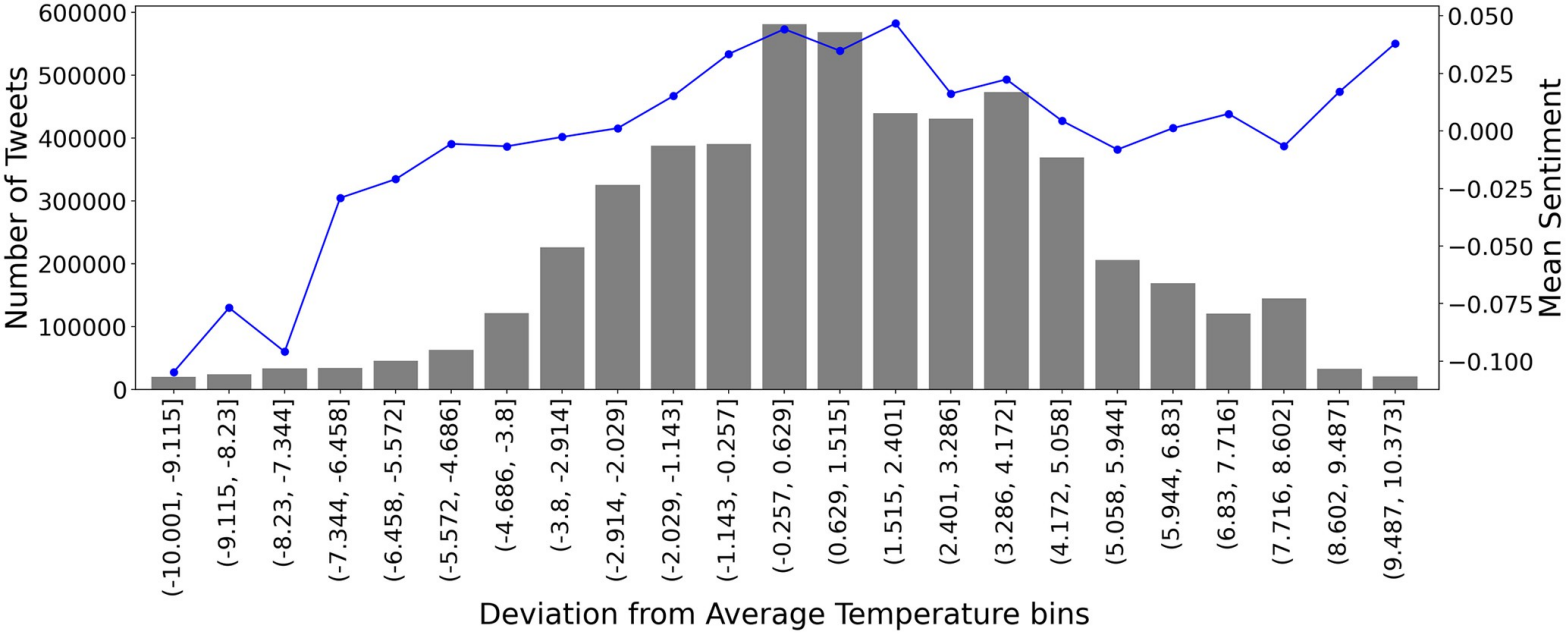
Deviation from average temperature after creating 50 equal bins versus the number of tweets (grey, left y-axis) and average sentiment (blue, right y-axis). Only bins between -10 and +10 temperature deviation are shown as the others contain significant few numbers of tweets, abnormal temperatures, and can be considered outliers.

Moving forward to whether any correlation between human sentiment and deviations from historic temperature exists, we proceed to create again 50 equally sized bins of temperature deviations. If we exclude temperature bins with very few points (outliers) we get Fig 8. The grey bars depict the total number of tweets in that particular bin and the blue lines show the mean sentiment of the bin. Observing this graph we extract the following insights. First, the sentiment (right y-axis) is negative until the temperature deviation of −2.914°*C* is reached (8th bin). Second, deviations from historic temperature are positively correlated with sentiment until +0.629°*C*. From +0.629°*C* to +2.401°*C* the sentiment is steadily positive. Then, it starts to slightly drop until +8.602°*C*, where it takes off again. Finally, it is evident that when temperatures are colder than the historic average, people tweet negatively about climate change, while when temperatures are warmer than the historic average, people tweet positively about climate change despite the fact that rising temperatures are an effect of climate change. The temperature deviations that people are the most positive are between −1.143°*C* and +2.401°*C*, being more forgiving to warmer temperatures.

**Fig 9:**
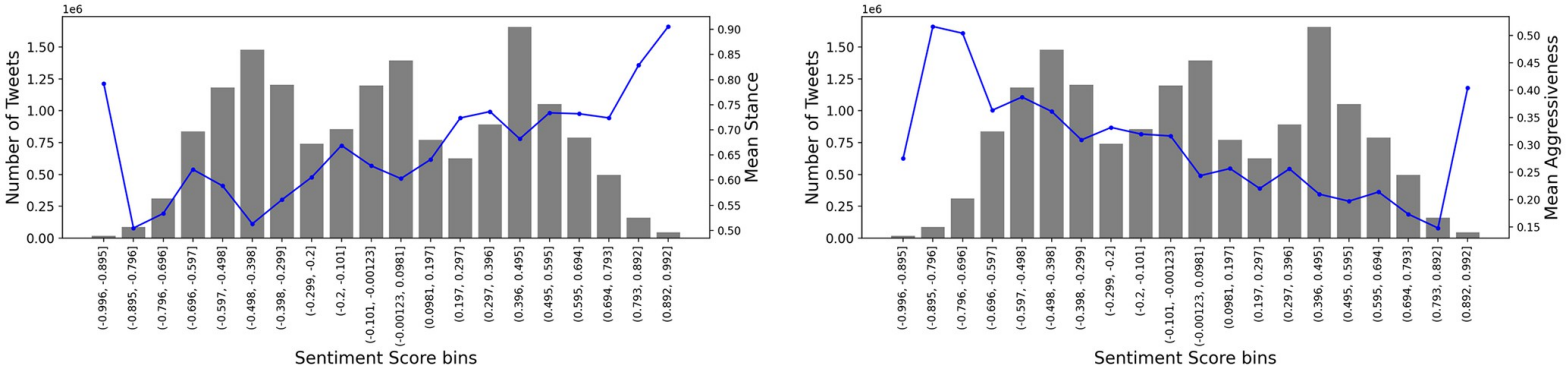
Relationship of sentiment and climate change stance (left graph) and aggressiveness (right graph). The sentiment was binned into 20 equal bins. In both graphs, the number of tweets in each bin are shown as grey bars (left y-axis). On the right y-axis and with blue colors the average climate change stance and average aggressiveness are displayed respectively.

We are also interested in how sentiment is related to stance and aggressiveness. In Fig 9, on the left graph the relationship of sentiment and stance is shown, while on the right graph, the relationship of sentiment and aggressiveness is shown. In both graphs, the x-axis is the sentiment in 20 equally sized bins from approximately -1 to +1. The mean stance is the average stance in that bin. If a tweet was classified as a climate change believer tweet, its score is 1. If it was classified as neutral its score is 0, and if it was classified as a climate change denier tweet, its score is -1. If we exclude the leftmost and rightmost bins which are very rare values as can be seen by the size of their bars, we get a clear positive correlation between sentiment and stance. There are three regions where the mean stance score reaches a local minimum, but this may be explained by the trinomial distribution of the sentiment scores. When there is a higher number of tweets, the mean stance score is slightly reduced. Nevertheless, the overall correlation is positive (+90%), suggesting that climate change deniers use more negativity in their tweets and climate change believers tweet more positively. The exact opposite behavior is observed from sentiment and aggressiveness if we remove the first and last bin, which are outliers. There is a clear negative correlation (-94%). The aggressiveness score is the average of the tweets inside each bin that are either aggressive (score of 1) or not (score of 0). As sentiment gets more positive, aggressiveness in tweets reduces.

**Fig 10:**
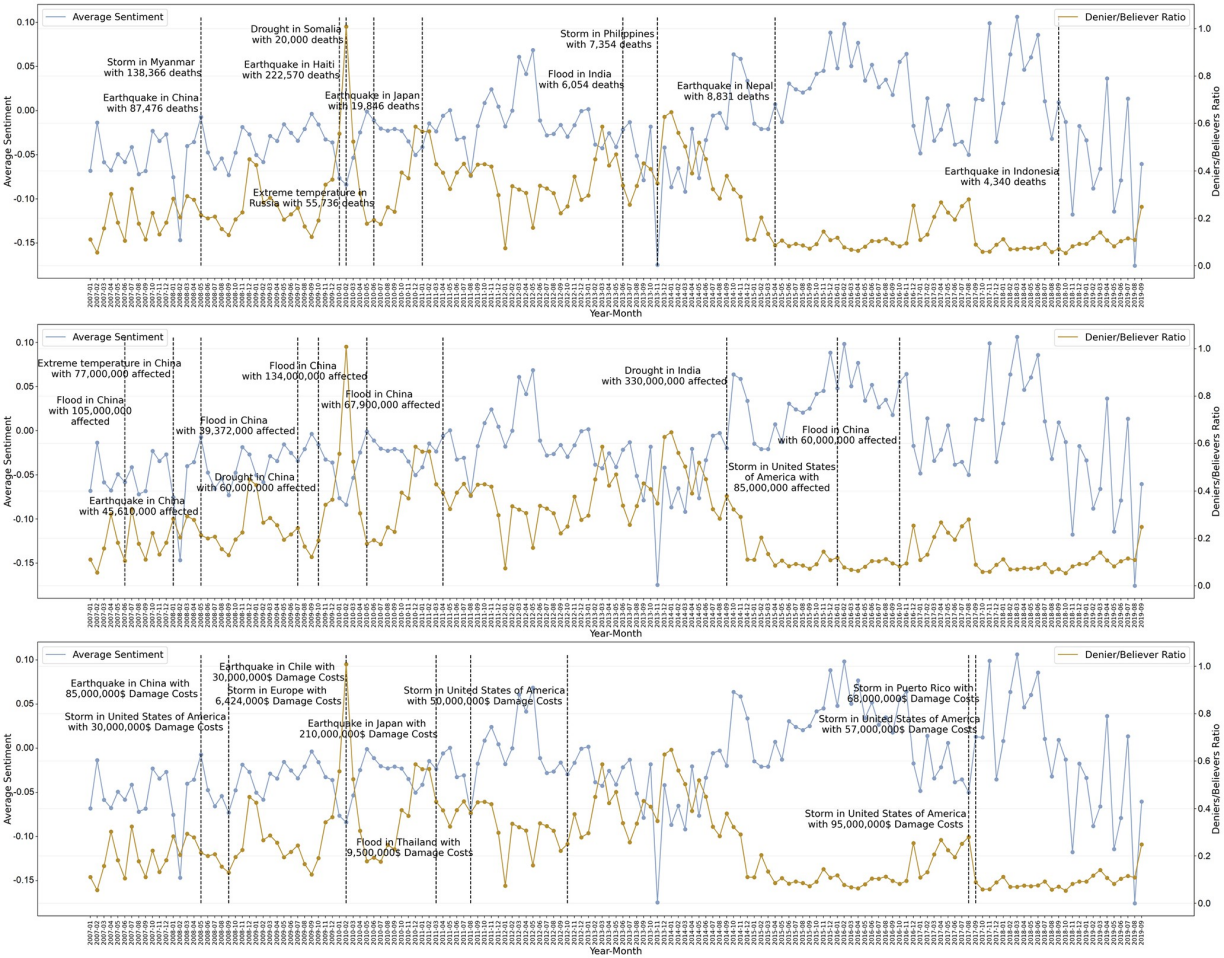
The ratio of climate change deniers/believers and sentiment over time. On the first plot, the ten disaster events with the most total deaths are marked. In the second plot, the ten disasters with the most affected are marked. In the third plot, the ten disaster events with the most damage costs are shown.

The last question that interests us is whether there are correlations between environmental disaster events and the denier/believer ratio or sentiment. In Fig 10 we examine three different aspects of disastrous events. The first concerns the events with the most total deaths. The top ten events are shown on the first graph. The time of the event, the type of the event, the location, and the number of deaths are available. Using the 4,913 events provided by the International Disaster Database we computed a correlation of 12% between total deaths and denier/believer ratio and -6% between total deaths and sentiment. In the same manner, the top ten events with the most affected people are shown on the second graph. The correlation between total affected from all events of the database and denier/believer ratio was found to be -6%, while the correlation with sentiment was -2%. The final aspect of a disaster is the damage costs it resulted. The third graph shows the ten most costly disaster events and the overall correlation is 6% with denier/believer ratio and 2% with sentiment.

No clear patterns are observed and the correlations are very low. The highest correlation is with total deaths (12% for denier/believer ratio and -6% for sentiment) suggesting that climate change deniers are triggered slightly more when human lives are lost from disaster events and the sentiment is slightly negative. Overall, we conclude that the stance of humans on man-made climate change and the sentiment are very low correlated —if not correlated at all?— with distinct disaster event features like total deaths, total affected, and damages costs. More complex interactions between disaster event characteristics, media coverage, the popularity of events, and other factors should be considered.

### Climate change discussed topics

In this section, we will answer two research questions: a) What topics are discussed in climate change/global warming discourses on Twitter? b) Is there any correlation between each of the discovered topics and the climate change stance, sentiment, and aggressiveness?

**Fig 11:**
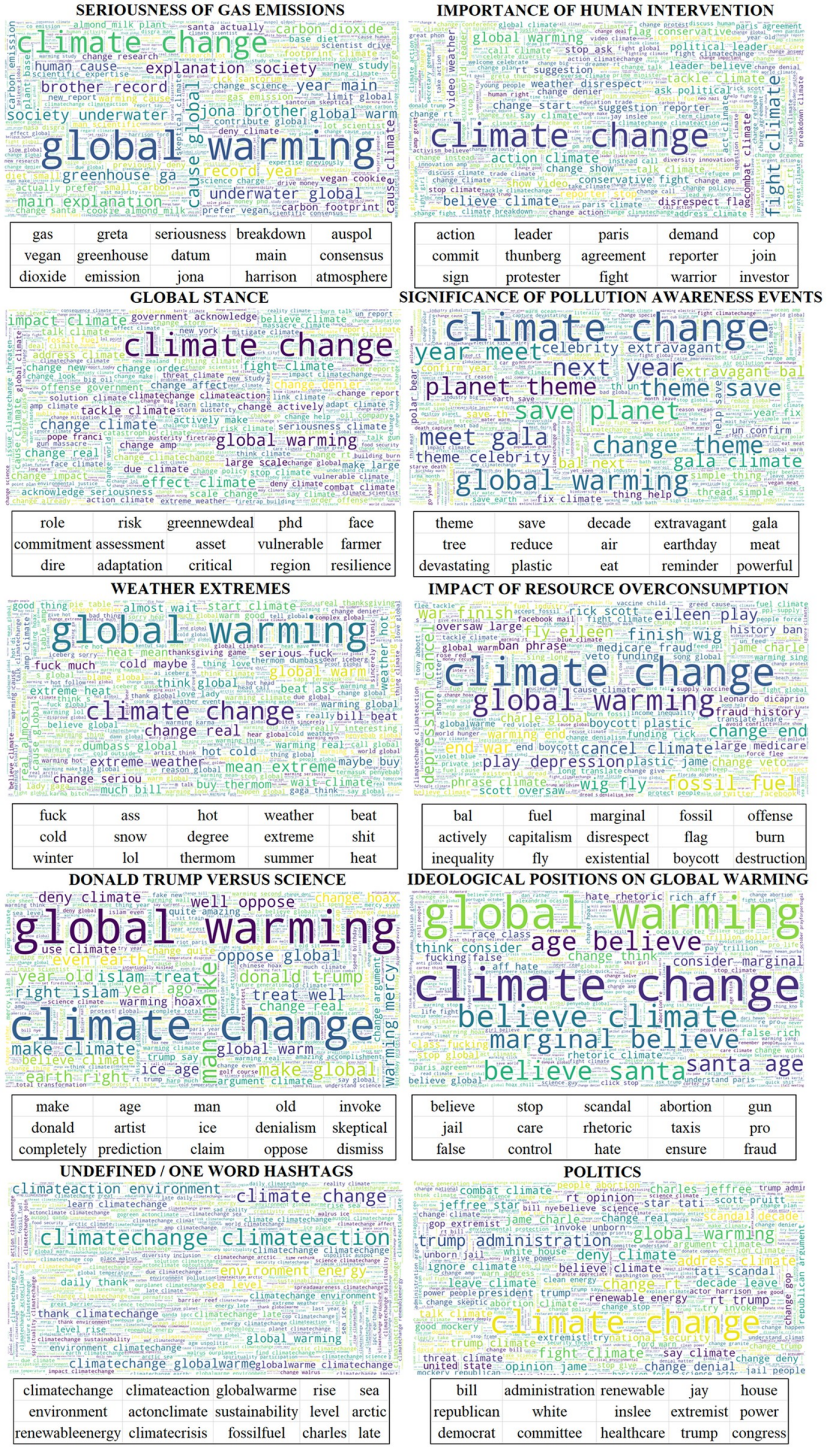
Ten topics discussed on Twitter about climate change. For each topic, there are available: its title, the word cloud with the most common 1,000 words and the 15 most unique words.

The ten topics discovered by the algorithm of the Climate Change Twitter Dataset are depicted in Fig 11. For each topic, there are present: the title of the topic, the word cloud of the 1,000 most common words in the topic, and the top-15 most unique words that were used to determine the topic.

The first topic is entitled ‘Seriousness of Gas Emissions’ and contains words like ‘gas’, ‘emissions’, ‘carbon’, ‘greenhouse’, ‘atmosphere’, and ‘dioxide’. ‘Global warming’ dominates ‘climate change’. The second topic is entitled ‘Importance of Human Intervention’ and with words like ‘action’, ‘leader’, ‘cop’, ‘Paris agreement’, ‘protestor’, and ‘fight’, refers to the actions people take to fight climate change. The third topic is named ‘Global Stance’ as it is a more general topic that includes words that suggest the stance on climate change of different people around the world. The fourth topic is named ‘Significance of Pollution Awareness Events’ as it contains words such as ‘theme’, ‘gala’, ‘save’, ‘earthday’, ‘reminder’, ‘plastic’, which indicate social events relative to climate change. The fifth topic is called ‘Weather Extremes’ and includes words such as ‘hot’, ‘weather’, ‘cold’, ‘snow’, ‘degree’, ‘extreme’, ‘winter’, ‘summer’, and ‘heat’. It also includes some profanity words, and ‘global warming’ is much more present than ‘climate change’. The sixth topic is entitled ‘Impact of Resource Overconsumption’ and contains words such as ‘fuel’, ‘fossil’, ‘offense’, ‘capitalism’, ‘burn’, ‘boycott’ and ‘destruction’. The seventh topic is about ‘Donald Trump versus Science’. The most unique words describing it are ‘age’, ‘man’, ‘old’, ‘Donald’, ‘denialism’, ‘skeptical’, ‘claim’ and ‘oppose’. The eighth topic is called ‘Ideological Positions on Global Warming’ with words like ‘believe’, ‘stop’, ‘scandal’, ‘gun’, ‘jail’, ‘care’, and ‘hate’. The ninth topic is an outlier one. The algorithm matched mainly words that are one-word hashtags like ‘climatechange’, ‘climateaction’, and ‘renewableenergy’. The tenth and last topic is about ‘Politics’. Its representative words are ‘administration’, ‘house’, ‘republican’, ‘white’, ‘extremist’, ‘power’, ‘democrat’, ‘committee’, ‘trump’, and ‘congress’.

**Fig 12:**
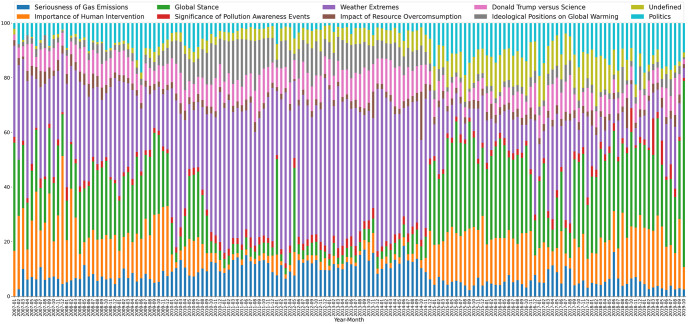
Topic percentage participation per month.

Table 2 presents per-topic results on different dimensions of the dataset, namely, stance classification (Denier/Believer Ratio and correlation with Denier/Believer Ratio), gender (Female/Male Ratio), sentiment (Mean Sentiment and Correlation with Mean Sentiment), and text aggressiveness (Mean Aggressiveness). Fig 12 shows for each month what is the percentage participation of each topic in the tweets posted in that month.

**Table 2:** Denier/believer ratio, female/male ratio, mean sentiment, and average text aggressiveness per topic.

Topic	Seriousness of Gas Emissions	Importance of Human Intervention	Global Stance	Significance of Pollution Awareness Events	Weather Extremes	Impact of Resource Overconsumption	Donald Trump versus Science	Ideological Positions on Global Warming	Politics
Denier/Believer Ratio	0.1878	0.0577	0.0411	0.0341	0.2636	0.1008	0.3996	0.2214	0.0828
Corr. with Denier/Believer Ratio	0.52	-0.35	-0.53	0.01	0.52	-0.10	0.34	0.54	-0.53
Female/Male Ratio	0.4346	0.4638	0.4544	0.6419	0.5034	0.5462	0.4087	0.5553	0.5043
Mean Sentiment	-0.0768	0.0798	0.0995	-0.0655	-0.1273	-0.0811	-0.1977	-0.2631	-0.096
Corr. with Mean Sentiment	0.17	0.49	0.76	0.15	0.35	0.44	0.13	0.05	0.43
Mean Aggressiveness	0.2622	0.2871	0.2367	0.1594	0.2432	0.3181	0.4029	0.3488	0.4339

The topic ‘Seriousness of Gas Emissions’ is constantly participating about 10% to 20% on the total tweets until December 2014, where it fades under 10%. It is highly positively correlated (0.52) with the denier/believer ratio, meaning that climate change deniers spark conversations about this topic. The total denier/believer ratio is 0.1878, which, comparing it to the other topics, is a high number favoring deniers. The mean sentiment is -0.0768, which indicates negative emotions, and the mean aggressiveness is 0.2622, which is relatively low. The female/male ratio is 0.4346 showing that males talk more about gas emissions.

The topics ‘Importance of Human Intervention’ and ‘Global Stance’ have similar characteristics. They are both almost absent between October 2010 and November 2014 but participate about 20%-30% outside of this range. They have a negative correlation of -0.35 and -0.53 respectively with the denier/believer ratio, indicating that climate change believers post more tweets regarding fighting climate change. Both topics are mainly discussed by males, have a very low total denier/believer ratio, low aggressiveness, and present the most positive sentiment across all topics (+0.0798 and +0.0995) respectively.

The topics ‘Significance of Pollution Awareness Events’ and ‘Impact of Resource Overconsumption’ are constantly present with a low share of about 5%, without any volatility, except some peaks at the last months of the dataset for the first, and a peak for the latter at October 2014. They are also uncorrelated (0.01 and -0.10 respectively) and have a low total denier/believer ratio (0.0341 and 0.1008). Both topics and especially the first are discussed more by females. Their sentiment is negative and their most notable difference lies in the text aggressiveness. The first has the lowest aggressiveness of all topics, while the latter has twice its aggressiveness.

The topic ‘Weather Extremes’ has a high share of percentage of the order of 30% to 60% with two gaps between January 2015 and October 2016, and March 2018 to October 2019. It is highly positively correlated (0.52) with denier/believer ratio, indicating that climate change deniers tweet more about extreme weather events. The total denier/believer ratio is 0.2636, which is the second-highest, while the topic is discussed equally by both females and males. There is a significant low sentiment score of -0.1273 and a relatively low aggressiveness.

The topic ‘Donald Trump versus Science’ has about 10% percentage share during his presidency, but also 5% to 12.5% outside his presidency. It is positively correlated (0.34) with the climate change denier/believer ratio showing the denialism that exists in its tweets. It has also a higher total denier/believer ratio (0.3996), a lower female/male ratio (0.4087), the second most negative sentiment (-0.1977) and it is the second most aggressive (0.4029).

The topic ‘Ideological Positions on Global Warming’ is discussed more by females than males, and is present mainly between January 2010 and October 2015. It has a correlation of 0.54 with denier/believer ratio and a total denier/believer ratio of 0.2214 meaning that deniers are more prone to this topic. Its aggressiveness score is very high (0.3488) and it tops every other topic in negativeness (-0.2631).

The topic ‘Politics’ starts to increase its volume since January 2015 with a mean participation of 15% and a correlation of -0.53 suggesting that climate change believers are the ones that most define this topic. It has a low total denier/believer ratio (0.0828) and is evenly discussed by females and males. Its sentiment is negative (-0.096) and its tweets are the most aggressive among all topics, with an impressive high aggressiveness score of 0.4339.

Climate change is a politicized issue and it is mainly communicated with negative sentiment, aggressive language, and denialism. People are expressing their concerns, especially when witnessing extreme weather and reprove actions responsible for climate change, like gas emissions and resource overconsumption. However, there are many discussions that point out the importance of human intervention to fight climate change. The global stance could be considered optimistic, as actions are being taken through events to raise awareness of this phenomenon.

## Conclusion

This study explores climate change using the Twitter social media platform. It uses a comprehensive dataset in both time and space, but also in volume. A total of 15 million tweets related to climate change and global warming are included, spanning over 13 years. Seven aspects (gender, stance, sentiment, aggressiveness, temperature, topics, and disasters) were studied and discussed in solitary and in combinations with others. We found that climate change believers differ from deniers, and identified locations on the world map where deniers are much more present. We revealed the relationship between temperature and denier/believer ratio, but also with the sentiment. The sentiment of climate change tweets over space and time was also explored and the correlation between human sentiment and deviations from historic temperature was investigated. In addition, we showed how are sentiment, stance, and aggressiveness related to climate change and inspected whether the sentiment and denier/believer ratio are correlated with disaster events based on total deaths, number of affected, and damage costs. Finally, we saw what topics are discussed in climate change and global warming discourses on Twitter and computed the correlation for each topic with each other aspect.

Results show that a major difference between believers and deniers is on the use of ‘global warming’ versus ‘climate change’ as believers use the term ‘global warming’ 5 times less often than ‘climate change’, while deniers favor ‘global warming’. Believers tweet more about taking actions to fight climate change, while deniers tweet more about global warming and extreme weather effects and their tweets have almost double aggressive language. Tweet geolocation revealed that regions with much more climate change deniers than world average include Canada, the United States of America, Cuba, South Africa, Japan, Eastern China, Australia, and Brazil. On the contrary, Europe, India, and Central Africa have low denier/believer ratios.

A negative correlation is evident between deviations from historic temperature and the denier/believer ratio. This indicates that people connect much more warm temperatures with man-made climate change than cold temperatures. In parallel, a positive correlation between denying that the source of climate change is humans and tweeting with negative sentiment is observed. Correlating sentiment and stance, the overall correlation is positive, suggesting that climate change deniers use more negativity in their tweets and climate change believers tweet more positively. The exact opposite behavior is observed from sentiment and aggressiveness, where correlation is negative. Overall, the stance of humans on man-made climate change and the sentiment exhibits limited correlation with the distinct disaster event features, like total deaths, total affected, and damages costs. More complex interactions between disaster event characteristics, media coverage, the popularity of events, and other factors should be considered.

Although most aspect interactions have been studied in this work, there are many more that potential researchers can explore and investigate in more depth. Complex interactions between climate change sentiment and external factors can be investigated in order to correlate extreme events with sentiment. Specific cases of extreme events or significant public environmental actions can be studied and the shift of human opinions can be tracked before, during, and after these events. Finally, these aspects can be studied in combination with the time-line that they occurred. For example, does sentiment and stance change over time and how is this change correlated with temperature and extreme events?
