# Correction: Exploring climate change on Twitter using seven aspects: Stance, sentiment, aggressiveness, temperature, gender, topics, and disasters

**DOI:** 10.1371/journal.pone.0293151

**Published:** 2023-10-12

**Authors:** Dimitrios Effrosynidis, Georgios Sylaios, Avi Arampatzis

There are errors in the Funding section. The correct Funding statement is: 1. Author Initials, DE, AA, GS 2. Grant number 101037643 3. Horizon 2020 4. https://ec.europa.eu/programmes/horizon2020/en/home 5. No, The funders had no role in study design, data collection and analysis, decision to publish or preparation of the manuscript.
